# Trends and Predictors of Large Tuberculosis Episodes in Cattle Herds in Ireland

**DOI:** 10.3389/fvets.2018.00086

**Published:** 2018-05-23

**Authors:** Tracy A. Clegg, Margaret Good, Martin Hayes, Anthony Duignan, Guy McGrath, Simon J. More

**Affiliations:** ^1^Centre for Veterinary Epidemiology and Risk Analysis, UCD School of Veterinary Medicine, University College Dublin, Dublin, Ireland; ^2^Independent Researcher and Private Consultant (previously affiliated with the Department of Agriculture, Food and the Marine, Dublin), Dun Laoghaire, Ireland; ^3^Department of Agriculture, Food and the Marine, Agriculture House, Dublin, Ireland

**Keywords:** bovine tuberculosis, *Mycobacterium bovis*, large episodes, Ireland, cattle

## Abstract

Persistence of bovine tuberculosis (bTB) in cattle is an important feature of *Mycobacterium bovis* infection, presenting either as herd recurrence or local persistence. One risk factor associated with the risk of recurrent episodes is the severity of a previous bTB episode (severity reflecting the number of bTB reactors identified during the episode). In this study, we have sought to identify predictors that can distinguish between small (less severe) and large (more severe) bTB episodes, and to describe nationally the severity of bTB episodes over time. The study included descriptive statistics of the proportion of episodes by severity from 2004 to 2015 and a case-control study. The case-control study population included all herds with at least one episode beginning in 2014 or 2015, with at least two full herd tests during the episode and a minimum herd-size of 60 animals. Case herds included study herds with at least 13 reactors whereas control herds had between 2 to 4 (inclusive) reactors during the first 2 tests of the episode. A logistic regression model was developed to identify risk factors associated with a large episode. Although there has been a general trend towards less severe herd bTB episodes in Ireland over time (2004–2015), the proportion of large episodes has remained relatively consistent. From the case-control study, the main predictors of a large episode were the year the episode started, increasing herd-size, previous exposure to bTB, increasing bTB incidence in the local area, an animal with a bTB lesion and a bTB episode in an associated herd. Herds that introduced more animals were more likely to have a smaller bTB episode, reflecting the reduced risk of within-herd transmission when an episode was due to an introduced infected bTB animal. Some of the risk factors identified in this study such as reactors in previous bTB episodes, herds with an associated herd undergoing a bTB episode, herds in high incidence areas etc. may help to target future policy measures to specific herds or animals for additional surveillance measures. This information has important policy implications.

## Introduction

Bovine tuberculosis (bTB) is a zoonotic disease caused predominantly by infection with *Mycobacterium bovis*. In Ireland, bTB is endemic in cattle and a test and slaughter eradication programme has been in place since 1954. The programme consists of testing every bovine in all herds annually using the Single Intradermal Comparative Tuberculin Test (SICTT) using Bovine Tuberculin PPD at 30,000 I.U./ml and Avian Tuberculin PPD at 25,000 I.U./ml, along with abattoir surveillance. The latter involves inspection of all bovine carcases at slaughter for tuberculous lesions by veterinary practitioners. When one or more positive animal is identified at the SICTT or at slaughter the herd is then “restricted” i.e., outward or inward movement of cattle is permitted only in accordance with EU Directive 64/432/EEC (1). The herd remains restricted until two consecutive negative herd tests, at 60 day intervals, are achieved. A bTB episode is defined as the full period when movement restrictions are imposed; that is, from initial detection of infected animals through to the final clearance test (generally the second consecutive negative herd test). Following “de-restriction” the herd is again free to trade, is then tested at 6 month intervals for two years and thereafter it returns to annual test intervals.

When the national programme first began, animal incidence was 17% (2) but has since declined to 0.26% in 2015 (More et al., submitted). In the latter year there were 3,823 new herd restrictions, giving a herd incidence rate of 3.37% (3). McGrath et al. (4) have highlighted an improving trend in many areas of Ireland. However, the improvements were highly heterogeneous and the overall decreasing trend was not uniform across the country. During 2003–12, the majority of herds had none or only one movement restriction due to bTB (hereafter referred to as a restriction), while 3.7% underwent two or more high risk restrictions and 0.9% had three or more, with a high risk restriction defined as at least 2 positive (reactors i.e., an animal removed under the bTB programme or lesion in a non-reactor) animals (5). Similar figures, in terms of positive animals, were found in Northern Ireland where 27% of herds contributed 56% of reactors between 2001 and 2003 (6). There are few published statistics, using Irish data, available that describe the severity of a restriction. A recent study (More et al., submitted), has looked at various measures of severity, duration and frequency of restrictions within the UK and Ireland, however, the herds included in the study had to meet certain criteria in order to make national comparisons. One aim of the current study will be to describe the severity of bTB restrictions within Ireland over time.

Persistence of bTB in cattle herds is an important feature of *M. bovis* infection, presenting either as herd recurrence or local persistence, and can be attributed to several sources such as residual infection, environmental infection (including wildlife), farm to farm transmission and the introduction of new infection following cattle movement (5). One risk factor identified as being associated with the risk of recurrent restrictions is the severity of a previous episode (7–12). Olea-Popelka et al. (7) found that herds with more than 8 reactors to the SICTT were nearly 3 times more likely to have another episode within 5 years compared with herds not previously infected. Similarly Wolfe et al. (8) found that cattle moved from a herd that had just had a bTB episode with at least 8 reactors were 1.8 times more likely to be bTB positive in the next 2 years when compared to animals moved from a non-infected herd. They also found that cattle moving from herds with 1 to 7 reactors had a non-significant increase (1.2 times) in the future risk of being positive. Wolfe et al. (9) looked at the future risk of a restriction for herds restricted in 2001 and found those with 1–5 standard reactors had a hazard ratio (HR) of a future restriction of 1.3 (95% CI: 1.1–1.4); those with more than 5 standard reactors had a HR of 1.6 (95% CI: 1.4–1.8) compared to herds with 0–1 standard reactors. Clegg et al. (12) found that herds with a more severe bTB episode in the past had higher odds of a future episode and that persistence continued for many years. In this study, herds with 2 or more reactors had a significantly increased risk of a future episode compared to those with 0 or 1 standard reactor. The risk decreased as time since the previous restriction increased but not significantly until at least 2 years prior to the current restriction.

Several studies have looked at the risk factors for predicting chronic episodes by considering either the length of the restriction period (6, 13, 14) or both restriction length and repeated episodes within herds (15). Griffin et al. (15) carried out a case-control study of chronic episodes identified as herds with recurrent episodes (≥2) or long duration episodes (>12 months) compared to herds free from bTB. The risk factors they identified for chronic episodes were: presence of badgers, nutritional factors, purchasing of cattle, and spreading of slurry. Doyle et al. (6) looked at longer duration episodes (lasting >1 year) and identified the following risk factors: location, previous history of bTB within the herd, severity of the index episode and presence of an animal with a lesion. Karolemeas et al. (14) compared prolonged (≥240 days) with non-prolonged episodes (<240 days). The main predictors that they identified were the confirmation status of an episode (i.e., an animal with visible lesion(s) at slaughter), cattle kept in covered yards, contact with non-contiguous domestic species on other farms, herd-size and movements during the episode into the herd. These were all associated with increased odds whereas salt lick use and movements in the previous year were associated with decreased odds. Reilly and Courtney (13) also compared transient (<6 months) and persistent (>6 months) episodes and found persistent episodes to be associated with herd type, silage storage, location and density of badgers.

The first aim of this study is to describe trends in the severity of bTB episodes in Ireland in terms of the number of infected animals that were detected per herd. A second aim is to identify predictors that can distinguish between small and large bTB episodes. Previous studies (6, 13–15) have concentrated on chronic herds by considering the duration of an episode. To the authors’ knowledge there are no other studies that have looked at predictors of large episodes in terms of the number of infected animals. Therefore, the objectives of this study were to identify risk factors associated with large bTB episodes in herds in comparison with smaller episodes, and to describe nationally the severity of bTB episodes occurring in Ireland.

## Material and Methods

### bTB Surveillance in Ireland

In Ireland, all cattle, aged over 6 weeks at the time of the test, or younger if introduced or in an infected herd, are tested annually for bovine tuberculosis using the SICTT in accordance with Annex B of Directive 64/432/EEC as amended section 2.2 (1). The SICTT involves the injection of bovine (potency 30,000 I.U./ml) and avian (potency 25,000 I.U./ml) tuberculin PPDs in the mid-third of the neck; the skin thickness at the site of the test is recorded at the time of injection and 72 h [±4 h] later. Any animal that displays clinical signs at the bovine injection site, such as oedema, exudative necrosis, heat and/or pain is positive and therefore a reactor. An animal with “a positive bovine reaction which is more than 4 mm greater than the avian reaction” is positive as per section 2.2.5.3.2 of the Directive (1) and deemed a “standard reactor”. When a standard reactor or an animal with clinical signs is identified, all animal movements are restricted until two clear consecutive SICTT tests are achieved on all animals within the herd, with at least a 60 day interval, the second of which must be carried out at a minimum of 4 months post removal of the last positive animal from the herd. An episode may also be triggered when an animal with a bTB lesion is detected at slaughter and movement restrictions and testing requirements are imposed in the same way as when a SICTT reactor is identified. In addition, “non-standard reactors” may also be identified during an episode, these are defined as all other animals removed under the bTB eradication programme during an episode with 2 or more standard reactors or bTB lesion animals cumulative, that have been defined as higher risk herds (12, 16). These “non-standard reactors” will include animals with “a positive or inconclusive bovine reaction which is from 1 to 4 mm greater than the avian reaction” i.e., standard inconclusive reactors and may include animals with a positive or inconclusive bovine reaction which is 0 to 2 mm less than the avian reaction i.e., severe interpretation inconclusive reactors, animals with a bovine reaction of 4 mm or more regardless of any avian reaction i.e., positive to the SIT (Single Intradermal Test), animals removed for epidemiological reasons by a Veterinary Inspector (VI) regardless of reaction at the bovine site or animals removed following the results of ancillary blood test(s), such as the interferon gamma (IFN-γ) assay (1, 16). In 2015, national policy in relation to strategic application of the IFN-γ assay in restricted herds was enhanced, with VIs instructed to sample cohorts of positive animals immediately after the first test of the episode in all herds with 4 or more animals already identified as reactors following the SICTT (16). It is acknowledged that the inclusion of non-standard reactors and particularly IFN- γ positive animals as reactor will have served to increase the number of reactor animals in episodes, however, in the Irish bTB eradication programme, such animals have a high probability of being bTB infected, of showing visible bTB lesions at slaughter and/or failing tests at a future date and thus their removal as reactors at the earliest possible stage under the programme is justified (17–21). Further details describing the protocol of managing bTB infected herds are described in the “Veterinary handbook for herd management in the bovine TB eradication programme” (16).

### Descriptive Analysis

The following descriptive statistics of herd-size and episode severity/duration were calculated from 2004 to 2015 inclusive:

- *Average herd-size over time*: Average size of the herd on the 31^st^ December each year was taken from statistics published by the Department of Agriculture, Food and Marine (DAFM; https://www.agriculture.gov.ie/animalhealthwelfare/animalidentificationmovement/cattle). The average herd-size at the start of each episode beginning within the respective year was estimated, based on the first full herd-test during the episode.
- *Number of bTB reactors during an episode by year the episode ended*: The number of SICTT reactors or animals with a bTB lesion at slaughter, for restrictions ending during the year of interest was calculated. For episodes starting after an animal with a lesion was detected at slaughter, it is assumed that a single animal with a bTB lesion triggered the episode.
- *Number of standard reactors/non-standard reactors at the start of an episode*: The number of standard/non-standard reactors detected on the first test during the episode (a full-herd test or if the first test was a part-herd test then the reactors identified on the part-herd test and at the next first full-herd test). Note a part-herd test may occur when only part of the herd is tested such as when conducting pre-movement testing or re-testing one or more animal(s) that were inconclusive at the previous test.

### Case-Control Study Population

The following criteria were used to identify herds eligible for consideration as either case or control herds: all herds with at least one episode beginning in either 2014 or 2015, with at least two full herd tests whilst restricted and before the end of 2015, and a minimum herd-size of 60 animals (this was the average herd-size in Ireland in 2015).

*Case herds* included all of the eligible herds with at least 13 reactors during the first 2 tests of the episode (unless the initial test was a part herd test, in which case the first 3 tests were used). A threshold of 13 reactors was chosen to represent a large episode, this being the top 5% of the distribution of the total number of reactors per herd within the first 2 tests of the episode during 2014/2015.

*Control herds* representing a small episode, included all of the eligible herds with between 2 to 4 (inclusive) reactors during the first 2 tests of the episode.

The study herds include both the case and control herds. For herds with more than one eligible episode, only the first episode was include in the study.

### Estimated Sample Size Needed for a Case-Control Study

The assumed exposure was whether the herd ever had a previous episode. An estimated sample size was based on 60% exposure in control herds (12), 95% CI, 80% power and an odds ratio (OR) of 1.9 for a future episode for a herd restricted in the last 5 years compared to those not restricted. The estimated sample size per group was 173.

### Risk Factors

The outcome measure was whether the herd was a case or a control herd. The following risk factors were considered in the analysis:

- Year the episode started (*epiyear*)
- Herd-size at the initial test of the episode (*herd_size*)
- Herd type (*herd_type*)
- Breeding herd (or non-breeding) (*breeding*)
- Test type (i.e., reason for testing the herd) at the initial episode test (annual test/(voluntary) pre-movement tests; re-test of an inconclusive reactor; forward trace of high risk animal(s) test; next test post de-restriction (i.e., the test 6 months following de-restriction); test of a herd contiguous to a restricted herd; test of a herd with an animal with a lesion found at slaughter) (*test_type*)
- Lesion present in one or more reactors, or the episode began with the detection of a lesion in an animal at slaughter (*lesion_present*)
- Previous history of bTB:
  - No. of previous episodes in last 10 years *(num_epi10yrs)*
  - Interval since last episode *(timesincelastepi)*
  - Number of reactors/standard reactors, reactors with a lesion or non-reactors with a lesion at the previous episodes *(prev_rct, prev_sdrct, prev_rxles, prev_facles)*
- Introductions: number of animals introduced into the herd in the current year/previous 3 years (*broughtin_currentyr, broughtin_3 years*)
- Ratio of introduced animals to herd-size at the initial episode test (*broughtin_hs_ratio)*
- Was the tester at the episode test the same as the tester at the previous test? *(same_prev_vet*)
- Was the tester at the episode test the same as the tester at the start of the previous episode? (*samebdvet*: 0 = not same tester, 1 = same tester, 2 = no previous episode)
- Average age of reactors and max age of reactors. Note for 9 animals born prior to 1996 the date of birth was not recorded and these animals were assigned an age of 19 (*mean_age, max_age*).
- Any current reactors present during the last previous episode in the same herd (*present_prebdown*)
- Any current reactors that were in the same age category (i.e., calves, heifers, cows, steers, bulls) as reactors at the previous episode in the same herd (*present_samegp_prebdown*)
- Any current reactors present during any previous episode in the same herd (*ever_prebdown*)
- Herd expanding? (% change in herd-size since previous year?) (*herd_expansion*)
- No. of fragments of land assigned to the herd (*fragment*)
- No. of neighbouring farms within 25, 150 or 500 m (*num_contigherds25, num_contigherds150, num_contigherds500*)
- Badgers: No. of badgers captured per year for previous 10, 5, 3 or 1 years (up to the year the restriction started but excluding the year the restriction started) within 25 m, 500 m or 1 km of the land fragment including area within the fragment *(bad25_10y, bad25_5y, bad25_3y, bad25_1y, bad500_10y, bad500_5y, bad500_3y, bad500_1y, bad1km_10y, bad1km_5y, bad1km_3y, bad1km_1y)*
- Geographical risk: Standard reactors per km^2^ in the previous year or 1–3 years (*rr_1 year, rr_3* years)
- Associated herd with an episode in the same year/previous year (*ass_epi*).

### Logistic Regression Model

Initially each of the risk factors listed above were tested in a univariable logistic regression model developed to model the probability of a herd being a case or a control herd. Risk factors that were significant in the univariable model (*p* ≤ 0.20) were considered for inclusion in a multivariable model. A backward selection procedure was used to eliminate risk factors from the multivariable model based on a likelihood ratio test (*p* ≥ 0.050). All variables with a p-value ≤ 0.20 in the univariable analysis were tested for collinearity to ensure a variance inflation factor (VIF) of <10 before being offered to the multivariable model. For risk factors with more than one measurement (e.g., contiguous herds within 25, 150 or 500 m), the appropriate measurement included in the multivariable model was based on the lowest AIC (Akaike Information Criteria). A plot of continuous variables against the log odds of the outcome and the variable in question was used to determine whether to include the variable as continuous or whether to transform the variable or include as a categorical variable (based on the quintiles of the variable). Interactions that were considered in the multivariable model were 2-way interactions between herd-type and herd-size; herd-type and reactor age. An assessment of the goodness-of-fit of the model was based on the Hosmer-Lemeshow test, the discriminatory ability of the model was assessed using the Area Under the ROC Curve (AUC) (22) and outliers were examined using influence statistics.

## Results

### Descriptive Analysis

**Figure 1:**
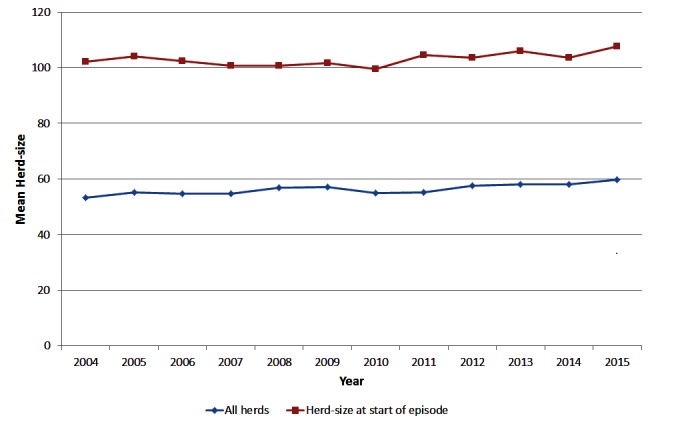
Mean herd-size of all herds in Ireland and for herds with a bTB episode starting in the year of interest, during 2004 to 2015.

The average size of herds in Ireland has been steadily increasing over time (Figure 1) from a herd-size of 53 animals in 2004 to 60 animals in 2015. For herds that had a bTB episode, the average herd-size at the start of the episode was considerably higher than for all herds nationally, increasing from 102 animals in 2004 to 107 animals in 2015, reflecting the higher risk of bTB in larger herds.

**Figure 2:**
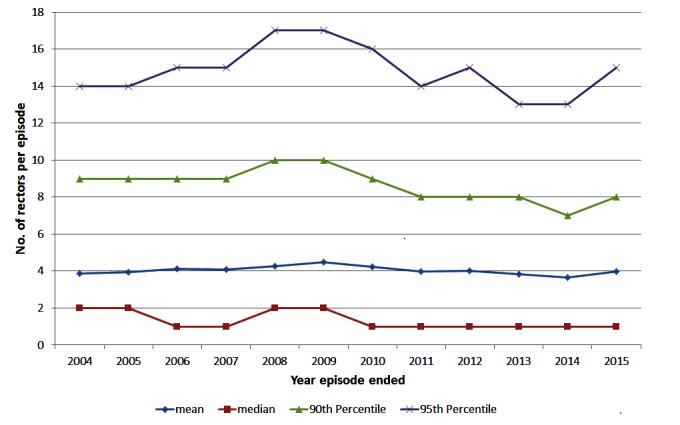
The number (mean, median, 90^th^ and 95^th^ percentile) of reactors (standard/non-standard and animals with a bTB lesion at slaughter) in herds with a bTB episode ending in the year of interest, during 2004 to 2015 in Ireland.

**Figure 3:**
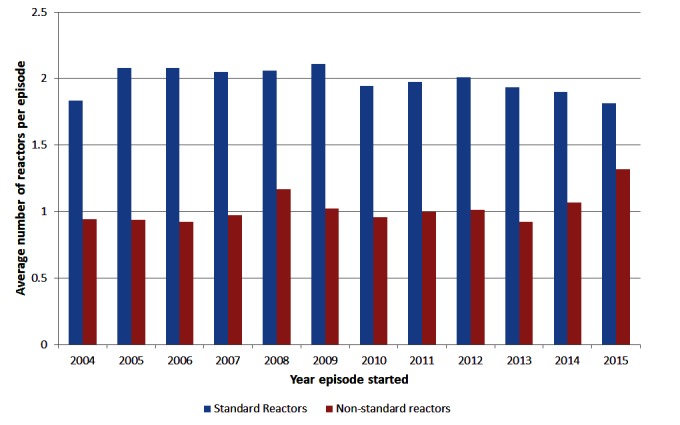
Mean number of standard and non-standard reactors at the first test during a bTB episode, for episodes in Ireland starting each year.

Among herds with episodes ending during each year of interest, the mean size of episodes has stayed relatively constant over time at around 4 reactors (Figure 2). The 95^th^ percentile of episode sizes increased to 17 reactors in 2008/2009 then decreased to 13 reactors in 2013/2014 with a slight increase in 2015 to 15 reactors (Figure 2). There was a significant (chi-square test *p* < 0.001) change in the proportion of restricted herds by severity of the episode over time (Table 1). The proportion of episodes that only involved 1 reactor/lesion has increased from 48.6% in 2004 to 57.8% in 2015 (Table 1). The proportion of herds having large episodes (≥13 reactors) peaked in 2008 at 7.4% and was the lowest in 2013 at 5.0%. There were more standard reactors compared to non-standard reactors, on average, at the start of an episode with between 1.81 and 2.11 standard reactors compared to between 0.92 and 1.3 non-standard reactors (Figure 3). Between 2014 and 2015, there was a decrease in the mean number of standard reactors at the start of an episode, but an increase in the mean number of non-standard reactors.

**Table 1:** Percentage of bTB episodes in Ireland by number of reactors detected during the episode (episode severity) and year that the episode ended.

Year episode ended	Number of reactors*				No. of episodes	No. of herds
	1	2–4	5–12	≥13		
2004	48.6	30.5	15.0	5.9	6474	6397
2005	49.5	28.7	15.9	5.8	6031	5960
2006	50.9	26.5	16.0	6.6	6025	5921
2007	52.9	26.2	14.0	6.9	6083	5964
2008	49.1	28.9	14.7	7.4	6204	6102
2009	48.0	28.4	16.3	7.3	5749	5640
2010	51.1	28.1	13.8	7.0	5061	4990
2011	52.3	29.3	12.8	5.6	4318	4261
2012	50.2	29.2	14.3	6.2	4247	4188
2013	52.8	28.4	13.8	5.0	4016	3939
2014	57.0	26.3	11.4	5.3	4057	3981
2015	57.8	25.2	11.0	6.0	3577	3500

*Number of reactors includes standard reactors, non-standard reactors and animals with a lesion at slaughter (assume 1 animal with a lesion identified at slaughter).

### Case-Control Study Population

A total of 321 herds met the case definition criteria and 996 herds met the definition of a control herd, giving a total of 1317 study herds. Of these, 722 study herds had episodes that began in 2014 (of which 164 (22.7%) were case herds) and 595 study herds had episodes that began in 2015 (of which 157 (26.4%) were case herds) (Table 2). Of the case herds, 57% were dairy, 34% suckler and 6% beef compared to 55%, 30% and 12% among control herds respectively. Control herds had a median number of 2 reactors per episode (range from 2 to 4) and case herds had a median of 22 reactors (range from 13 to 294). Of the control herds, 6.6% had been tested using the IFN-γ assay compared to 57.9% of case herds. In 2015 a higher proportion (70.1%) of case herds were tested with the IFN-γ assay compared to case herds in 2014 (46.3%). Of the case herds, 54 (16.8%) had <13 SICTT reactors, the remainder were positive to the IFN-γ assay. These 54 herds had a median of 9 SICTT reactors and 43 of them were in episodes starting in 2015.

**Table 2:** Percentage of herds in Ireland with a large bTB episode (≥13 reactors in the first 2 full herd tests) commencing in 2014–15, by significant (*p* < 0.2) risk factors.

Risk factor	Categories	No. of herds with an episode	Large episodes		p-value
			Number	%	
Year episode started(*epiyear*)	2014	722	164	22.7	0.123
	2015	595	157	26.4	
Herd size(*herd_size*)	60–84	267	47	17.6	0.002
	85–113	262	61	23.3	
	114–154	259	65	25.1	
	155–223	266	62	23.3	
	>223	263	86	32.7	
Herd type(*herd_type*)	Beef	138	21	15.2	0.034
	Dairy	729	183	25.1	
	Suckler	410	109	26.6	
	Other	40	8	20.0	
Breeding herd(*breeding*)	Yes	1167	301	25.8	<0.001
	No	150	20	13.3	
Episode test type(*test_type*)	Annual/premovement test	566	113	20.0	<0.001
	Inconclusive reactor re-test	73	6	8.2	
	Forward trace of a high risk animal(s) test	179	62	34.6	
	Post de-restriction test	119	26	21.8	
	Contiguous herd test	244	66	27.0	
	Lesion at slaughter: herd test	136	48	35.3	
Lesion present in one or more reactors/animal at slaughter (*lesion_present*)	None	465	30	6.5	<0.001
	One or more	852	291	34.2	
Number of previous episodes in last 10 years (*num_epi10yrs*)	0	409	100	24.4	0.010
	1	391	84	21.5	
	2	265	81	30.6	
	3	136	38	27.9	
	>3	116	18	15.5	
Ratio of introduced animals to herd-size at the initial episode test (*broughtin_hs_ratio*)	0	277	75	27.1	0.079
	0.001–0.0167	249	71	28.5	
	0.0168–0.0706	264	63	23.9	
	0.0707–0.2194	264	63	23.9	
	>0.2194	263	49	18.6	
Same tester at most-recent previous episode (*samebdvet*)	Different tester	777	175	22.5	0.151
	Same tester	401	106	26.4	
	No previous episode	139	40	28.8	
Max. age of reactors (years) (*max_age*)	0–3.5	263	22	8.4	<0.001
	3.6–6.0	264	17	6.4	
	6.1–8.4	263	45	17.1	
	8.5–11.2	263	76	28.9	
	>11.2	264	161	61.0	
Any reactor present at the most-recent previous episode	None	676	119	17.6	<0.001
	One or more	641	202	31.5	
Any reactor present at the most-recent previous episode in same age class (*present_samegp_prebdown*)	None	868	180	20.7	<0.001
	One or more	449	141	31.4	
Any reactor ever present during any previous episode (*ever_prebdown*)	None	535	73	13.6	<0.001
	One or more	782	248	31.7	
No. of farm fragments (*fragment*)	1–2	196	41	20.9	0.126
	3–4	351	95	27.1	
	5–6	287	57	19.9	
	7–8	200	52	26.0	
	>8	279	75	26.9	
No. of badgers captured over previous 10 years within 1 km of the farm (*bad1km_10y*)	0–2	249	51	20.5	0.011
	3–9	270	50	18.5	
	10–17	283	85	30.0	
	18–28	254	66	26.0	
	>28	257	68	26.5	
Geographical risk in the previous year : Reactors per km^2^ (*rr_lyr*)	0–0.001	263	25	9.5	<0.001
	0.001–0.002	263	46	17.5	
	0.002–0.004	263	71	27.0	
	0.004–0.007	263	79	30.0	
	>0.007	263	100	38.0	
Associated herd with an episode in current/previous year (*ass_epi*)	None	1270	297	23.4	<0.001
	One or more	47	24	51.1	

### Logistic Regression Model

Risk factors that had a p-value < 0.2 in the univariable analysis are presented in Table 2. When there was more than one variable used to measure the same risk factor, the one with the lowest AIC in the univariable model was included in Table 2. At the univariable level, there were significant differences (*p* < 0.05) in the number of case herds by: herd-size, herd-type, breeding herd, episode test type, lesion present in one or more reactors, number of previous episodes in the last 10 years, maximum age of reactors, any reactors present at previous episodes, badger density, geographical risk and episodes in associated herds.

Variables representing a previous episode i.e., *present_prebdown, present_samegp_prebdown, ever_prebdown* were all correlated. The variable: “reactors ever in a previous episode” (*ever_prebdown*) had the lowest AIC and was included in the multivariable model.

Herd-size as a log transformed continuous variable gave the best fit at the univariable level based on the AIC and a plot against the log odds of being a case and was included in the multivariable model. Similarly the log of the area relative risk gave the best fit at the univariable level. Herd-type and breeding herds were correlated, herd-type had the lower AIC and was considered for inclusion in the multivariable model. Out of all the measures of badger density, the number of badgers within 1 km of the farm over 10 years had the lowest AIC.

The final multivariable model included the variables: year the episode started; log of herd-size; episode test type; log of the area relative risk in the previous year; whether any reactor(s) had ever been in a previous episode, whether an associated herd had an episode in the same/previous year, the ratio of the number of animals brought-in: herd-size and whether there was a lesion present in a reactor or an animal at slaughter (Table 3). For herd-size and area relative risk, the log of the variable was included in the final model. The inter-quartile range for herd-size in this study was approximately 100 animals, an increase in herd-size by 100 would mean a 40 times increase in the odds of having a large episode (OR: 40.3, 95% CI: 11.4–145.0). Similarly the inter-quartile range for the area relative risk was approximately 0.004 reactors per km^2^. An increase in the area relative risk by 0.004 reactors per km^2^ would more than double the odds of having a large episode (OR: 2.7, 95% CI: 2.1–3.4).

**Table 3:** Parameter estimates from the logistic regression model of the probability of a large bTB episode (≥13 reactors) in Irish herds during 2014–15.

		OR	Lower	Upper	P-value
Year episode started (*epiyear*)	2014	Referent			.
	2015	1.48	1.11	1.99	0.009
Log Herd-size (*herd_size*)		2.23	1.70	2.95	<0.001
Log geographical risk in the previous year: Reactors per km^2 ^(*rr_lyr*)		2.03	1.70	2.43	<0.001
Associated herd with an episode in current/previous year (*ass_epi*)	No	Referent			.
	Yes	2.06	1.05	4.08	0.037
Any reactor ever present during any previous episode(*ever_prebdown*)	None	Referent			.
	One or more	2.64	1.90	3.69	<0.001
Ratio of introduced animals to herd-size at the initial episode test (*broughtin_hs_ratio*)	0	Referent			.
	0.001–0.0167	0.88	0.56	1.37	0.564
	0.0168–0.0706	0.67	0.42	1.04	0.076
	0.0707–0.2194	0.59	0.38	0.93	0.023
	>0.2194	0.51	0.32	0.81	0.005
Lesion present in one or more reactors or an animal at slaughter (*lesion_present*)	None	Referent			
	One or more	6.63	4.41	10.30	<0.001
Episode test type (*test_type*)	Annual/premovement test	Referent			.
	Inconclusive reactor re-test	0.45	0.16	1.07	0.095
	Forward trace of a high risk animal(s) test	1.31	0.85	2.02	0.217
	Post de-restriction test	0.56	0.32	0.97	0.044
	Contiguous herd test	0.90	0.59	1.35	0.601
	Lesion at slaughter: herd test	1.02	0.64	1.61	0.945

Herds had significantly larger episodes (case herds) when they began in 2015, were larger herds, involved an animal with a bTB lesion, were in an area with a high relative bTB risk, had one or more reactor(s) present during a previous episode and/or had an associated herd with an episode in the current/previous year. Herds that introduced more animals relative to herd-size were significantly less likely to have a large episode and episodes that began with a post-derestriction test (i.e., at 6 months following a previous de-restriction) also had lower odds of a large episode compared to herds starting an episode at the annual test. The Hosmer-Lemeshow test (*p* = 0.290) and the residual analysis indicated no significant lack of fit, the AUC of 0.817 indicated an adequate discriminatory ability of the model.

The median age of reactors was also a significant variable and gave a better fitting model (Supplementary material, Table S1) than that in Table 3 (AIC: 1136.8 versus 1170.8). However, in control herds the median age was only based on a small number of reactors (2 to 4) and was very variable (see Figure S1) in these herds therefore this variable was excluded due to uncertainty regarding whether any observed differences were mainly due to small number of animals. Similarly, the maximum and minimum age of reactors was considered; however, due to the large variation in range (see Figures S2, S3 ) among the larger episodes, it was decided to exclude any age variables from the models.

A model using the variable: “number of episodes in the previous 10 years” was created by introducing this variable instead of the variable: “any reactor present in a previous episode” (Table S1). This model was not as good a “fit” to the data as the model in Table 3 (AIC 1193.5 versus 1170.8), however, this variable is informative regarding the previous history of herds with larger episodes. The odds of a large episode decreased once the number of episodes in the previous 10 years increased to more than 3.

## Discussion

### National Trends

In Ireland, the average size of an episode has remained relatively constant over the last 10 years at approx. 4 reactors per episode (Figure 2). The proportion of episodes with only 1 reactor has been increasing over time, whereas there has been a decrease in the proportion of most other sizes of episode over the last 10 years (Table 1). It is probable that a small proportion of the episodes with a single reactor are due to false positive reactions to the SICTT given the imperfect specificity of the test (23–25). As the national prevalence of bTB decreases, we would expect to see a higher proportion of singleton restrictions as the relative percentage of restrictions due to the decrease in true infection over time. However, the proportion of episodes with ≥13 reactors has remained fairly constant at 5.0 to 6.2% of episodes over the last 5 years (Table 1). The overall size of herds and the size of herds with a bTB episode have remained relatively constant reflecting that any improvements are unlikely to be due to changes in herd-size. Given the consistent proportion of larger episodes over time, it is important to identify any underlying risk factors.

### Residual Infection

This study identified a number of significant predictors of a large episode compared to small episodes with limited within-herd transmission. Some of these predictors are indicative of residual infection [that is infected but undetected cattle (5)] within the herd resulting in within-herd transmission prior to disclosure. One such predictor is whether an animal with a lesion was present within the episode. Episodes that included an animal with a lesion were more than six times as likely to result in a large bTB episode compared to episodes with no animal with a lesion. Karolemeas et al. (14) also found that an episode that was confirmed (following detection of a visible lesion or culture of *M. bovis* in one or more reactors) was a significant predictor for a prolonged episode. Similarly, Reilly and Courtenay (13) found 92% of persistent episodes (>6 months) were confirmed compared to only 63% of transient (<6 months) episodes. Episodes without any animals with a lesion may be a consequence of latent infection (26), or a less advanced stage of disease, each of which may not be detected by examination at slaughter. In the case of latent infection, within-herd transmission may follow subsequent to the reactivated infection in an animal. Evidence of reactivation in cattle comes from the Australian bTB eradication programme where infected cattle were detected in the absence of an external infection source [Cousins et al., (27) as cited in Karolemeas et al. (10)]. Within-herd transmission in herds where a lesioned animal had been detected at slaughter has been examined by Olea-Popelka et al. (28). They found that one risk factor for disclosure of additional animals was whether the animal with a lesion had been present in a previous bTB episode and the time the animal had spent in the study herd. In this study, a herd with a reactor that had been in a previous bTB episode had 2.6 times the risk of having a large episode compared to herds with no reactors in a previous bTB episode. Doyle et al. (6) also found previous history, measured as the total time restricted in the previous 5 years, was the best predictor of both long and recurrent episodes. Many studies have also found previous bTB history to be a predictor of bTB within a herd (2, 12) and for recurrence within a herd (7, 10, 11, 29). Animals that have been in a previous bTB episode were possibly missed at a previous SICTT, which may partly reflect the imperfect sensitivity of the SICTT, with a median value of 80% (range 52 to 100%) based on several studies (20, 25, 30, 31) and between 64.5 and 73.0% based on a Bayesian latent-class analysis of Irish data (32). The imperfect sensitivity will result in infected animals being missed by the SICTT and left in the herd with the possibility of subsequent within-herd transmission.

### Post-Derestriction Test and Number of Previous Episodes

Episodes that began at a post-derestriction test had significantly lower odds (OR: 0.56, 95% CI: 0.32–0.97) of being a large episode compared to episodes that began with an annual test. The post de-restriction test takes place 6 months following de-restriction of the herd and non-standard reactors are removed even if no standard reactor is present on this test i.e., these tests have the severe interpretation of the SICTT applied. The proportion of herds positive at the post-derestriction test in Ireland has been reasonably constant over time at around 12% between 1995 and 2009 (33) falling to 9.4% in 2015 (More et al., submitted). Infected animals detected at this test may plausibly reflect animals that have been missed in the previous episode.

Herds that previously had more than three bTB episodes in the previous 10 years (Table S1) also had lower odds of a large episode. It is likely that these herds have had more severe controls imposed such as an increased number of tests following previous episodes and a more severe interpretation level (16). Infected animals identified in the current episode are animals either previously missed or bought-in following previous episodes, therefore with limited within-herd transmission.

### Geographical Risk

Herds in areas with a high incidence of bTB were more likely to have a large episode reflecting the increased infection pressure within the locality. This has been found in many other studies that looked at both the occurrence (2) and recurrence (7, 9, 11) of bTB within herds. Doyle et al. (6) also found an increased risk of chronic episodes due to infection in the neighbourhood. One source of neighbourhood infection is infected wildlife, which in Ireland is mostly considered to be badgers (15, 34–36). White et al. (2) found an increased risk of bTB associated with herds at a distance of between 25 m and 1 km, the authors concluded that infected wildlife was the most likely explanation of this locality risk. Badger density in the vicinity of the study herds was examined in several different ways, including varying the distance from the farm and the number of years of culling. The best fitting predictor at the univariable level was the number of badgers culled within 1 km of a farm over 10 years; however, this was not significant within the final model. Farms that had culled 10–17 badgers had the highest proportion of large episodes, possibly reflecting an ongoing problem in the area.

### Herd-Size

Only herds that were above the national average herd-size of 60 animals were included in the study. However, the odds of a large episode still increased with increasing herd-size. The mean herd-size of restricted herds was larger than the mean of the national population of herds (Figure 1), reflecting the higher risk of these herds having an episode. In this study, the risk of a large episode increased with the log of the herd-size (Table 3). Of the largest herds (>223 animals), 32.7% experienced a large episode (≥13 reactors) compared to 17.6% of the smallest herds in the study (60–84 animals) (Table 2). Many studies [summarised by Skuce et al. (37)] have found an association between herd-size and the risk of bTB occurrence, others (6, 7, 9, 11) found an association with recurrence and two others (14, 38) with prolonged episodes. These higher risks to larger herds may be due to a number of factors such as the larger area of the farm which increases the risk of exposure to infected wildlife and infected neighbouring herds. In addition, as the herd-size increases there is an increasing risk that an infected animal may not be detected by the SICTT due to the imperfect test sensitivity, which therefore prolongs the episode allowing the potential for additional transmission of infection. In addition, intensive management of larger herds such as less attention to individual animals, has also been associated with an increased risk of a chronic episode (15).

### Year the Episode Started

The odds of a herd having a large episode were 1.5 times higher in 2015 compared to 2014. This could, at least partially, be attributed to the increased and more targeted use of IFN-γ in 2015 in episodes with at least 4 reactors. In 2015 a higher proportion of case herds were tested with the IFN-γ assay compared with 2014 case herds (70.1% versus 46.3%). In addition of the 54 herds that qualified as a case herd due to additional IFN-γ positives 80% of the episodes began in 2015. This is also reflected by the increase in non-standard reactors in 2015 (Figure 3). Prior to the enhanced policy instruction, VIs were recommended to sample animals from all episodes with at least 4 SICTT reactors, however, not all such episodes were subjected to sampling. The application of the IFN-γ test will have had the potential to remove infected animals, particularly those in the earlier stages of infection sooner. Gormley et al. (20) found animals that were SICTT negative/IFN-γ positive, were up to 9 times more likely to become SICTT positive when followed up for two more SICTT tests compared to SICTT negative/IFN-γ negative animals. Clegg et al. (17) looked at post-mortem results of animal that were negative to the SICTT and IFN-γ tested and slaughtered in the same year. In this study, the odds of an IFN-γ positive animal being positive at post-mortem was nearly five times higher compared to IFN-γ negative animals. Therefore, the increased use of the IFN-γ will initially be expected to give rise to larger episodes but should potentially reduce the risk of missing infected animals that could cause future recurrence and within-herd transmission.

### Introduced Cattle

The odds of a large episode decreased as the ratio of animals introduced: herd-size increased. This plausibly reflects episodes due to introduced animals tending to involve very little within-herd transmission. Reilly and Courtenay (13) looked at transient (<6 months) and persistent (>6 months) episodes in Great Britain and found variables associated with cattle purchase were important risk factors for transient episodes but not for persistent episodes. Karolemeas et al. (14) also found decreased odds of a prolonged episode associated with increasing number of cattle bought-in during the 12 months prior to the episode.

### Associated Herds

An associated herd is a herd that is linked to another for management or epidemiological reasons e.g., due to a family or partnership relationship with individuals managing different aspects of the farming livestock business/enterprise on separate holdings. Many larger herds tend to split animals into different production and epidemiological groups e.g., calf/heifer rearing/breeding separated from milking cows often with more than one herd number. Thus a large herd that has its animals spread between two herd numbers may therefore have split infected animals between herds prior to the commencement of an episode. The increased risk from an associated herd may also be representative of a contiguous risk since the animals in the associated herd may remain within the immediate neighbourhood and are often in much closer contact compared to contiguous herds due to shared management and risk factors. Associated herds are subjected to the same controls and restrictions when positive animals are detected in one or other which necessarily results in restriction and testing of associated herds in cases where the index herd had an episode.

### Methodological Issues

This study looked at restricted herds only i.e., the difference between a large and small episode as opposed to having/not having bTB. All herds had two full-herd tests within the study period to be included in the study. This rule was included so that herds with an ongoing episode towards the end of 2015 were excluded unless they had 2 full herd tests. The study results were, therefore, based on the number of reactors found at the beginning of an outbreak, reflecting risk factors for more “explosive” episodes with considerable within-herd transmission prior to detection. In GB, Karolemeas et al. (14) found that episodes with more reactors at the start were associated with longer episodes, although this was confounded with confirmation status. This is at odds with work from Northern Ireland where Doyle et al. (6) found that an increased number of reactors at the breakdown test were associated with reduced odds of a prolonged episode. Doyle et al. (6) speculated that the more severe the initial intervention, and therefore the more reactors identified, the faster the infection was cleared.

The significant effect of some variables such as the age variables may be an artefact of the number of reactors in the case and control herds. In the supplementary material, Figures S1–S3 show how the median, min and max age of reactors vary with the number of reactors within the episode. The same may also be true for the presence of a reactor with a lesion, since the sensitivity of the post-mortem test is thought to be lower than the SICTT (25) and the probability of detecting an animal with a lesion can vary by slaughterhouse (39); therefore there is a higher probability of finding a lesion when the sample size is larger. However, even if these variables are artefacts of the sample size the remaining variables were consistent across all of the models.

It was not possible to look at some of the risk factors identified in other studies such as silage storage, salt licks, nutrition etc. as such data are not available. More detailed case-control studies may be able to identify other risk factors that may be associated with larger episodes.

### Policy Implications

In Ireland, herds with more severe episodes (2 or more standard reactors or bTB lesion animals, cumulative) are designated as higher risk status and accordingly undergo more rigorous testing post de-restriction and must pass three tests at 6 month intervals before returning to default risk status. In Australia, during the bTB eradication programme, herds were placed under longer restriction controls and herds were not entirely free to trade until 8 years after the last infected animal was detected (40). Herds that have had a large episode have been shown to pose a risk of having another episode in the future (7, 9, 11, 12, 29). Future controls on these herds will need to be continually reassessed to look at whether additional measures are appropriate, such as maintaining the higher risk classification and rigorous testing of herds following a severe episode for longer periods after the episode has ended.

Some of the risk factors identified in this study such as reactors in previous episodes, herds with an associated herd undergoing an episode, herds in high incidence areas etc. may help to target future policy measures to specific herds or animals that could be targeted for additional surveillance measures. Additionally, further work is needed to assess whether the increased and focused use of the IFN-γ assay in herds experiencing a severe episode during 2015 has shortened the duration of the episode and/or reduced the risk of repeat episodes of bTB in these herds.

##  Conclusions

Although there has been a general trend towards less severe herd bTB episode in Ireland over time, the proportion of large episodes has remained relatively consistent. An understanding of the risk factors that influence these large episodes is important, to improve national controls. Based on the results from this study, the main predictors of a large episode were the year the episode started, increasing herd-size, previous exposure to bTB, increasing bTB incidence in the local area, an animal with a bTB lesion and a bTB episode in an associated herd. Herds that introduced more animals were more likely to have a smaller bTB episode, reflecting the reduced risk of within-herd transmission when an episode was due to a purchased infected bTB animal. This information has important policy implications. 

## Author contribution

MG and MH formulated the idea for the study. SM, TC, MG, MH and AD developed the study design. TC carried out the statistical analysis. GM prepared the geographical information. TC wrote the first draft. All authors contributed to the final draft.

## Conflict of Interest Statement

The authors declare that the research was conducted in the absence of any commercial or financial relationships that could be construed as a potential conflict of interest.

## Supplementary material

Table S1 Parameter estimates from two additional logistic regression models of the probability of a large (≥ 13 reactors) bTB episode in Ireland during 2014-15; (i) including the variables: median age of reactors and (ii) substituting the variable ‘any reactor ever present in a previous breakdown’ with ‘number of previous episodes in the last 10 years’.

Figure S1 Median age of reactors by the number of reactors in the breakdown.

Figure S2 Minimum age of reactors by the number of reactors in the breakdown.

Figure S3 Maximum age of reactors by the number of reactors in the breakdown.
