# Supplementary material for: Trends and Predictors of Large Tuberculosis Episodes in Cattle Herds in Ireland
Source: Front Vet Sci. 2018 May 23;5:86. doi: 10.3389/fvets.2018.00086 (PMC5974150; doi:10.3389/fvets.2018.00086)
Supplement: Table S1 — Parameter estimates from two additional logistic regression models of the probability of a large (≥ 13 reactors) bTB episode in Ireland during 2014-15; (i) including the variables: median age of reactors and (ii) substituting the variable ‘any reactor ever present in a previous breakdown’ with ‘number of previous episodes in the last 10 years’. [file Table1.DOCX]

Table S1: Parameter estimates from two additional logistic regression models of the probability of a large (≥ 13 reactors) bTB episode in Ireland during 2014-15; i) including the variables: median age of reactors and ii) substituting the variable ‘any reactor ever present in a previous breakdown’ with ‘number of previous episodes in the last 10 years’

|  |  | 1. Median age | | | |  | 1. number of episodes within last 10 years | | | |
| --- | --- | --- | --- | --- | --- | --- | --- | --- | --- | --- |
|  |  | OR | Lower | Upper | P-value |  | OR | Lower | Upper | P-value |
| Year episode started | 2014 | Referent |  |  | . |  | Referent |  |  | . |
|  | 2015 | 1.41 | 1.05 | 1.90 | 0.023 |  | 1.40 | 1.05 | 1.88 | 0.023 |
| Log Herd-size |  | 1.98 | 1.50 | 2.61 | <.0001 |  | 2.45 | 1.86 | 3.26 | <.0001 |
| Log area rel. risk in previous year | | 2.02 | 1.70 | 2.41 | <.0001 |  | 2.09 | 1.76 | 2.51 | <.0001 |
| Median age | <2.27 | Referent |  |  | . |  |  |  |  |  |
|  | 2.27-3.56 | 0.82 | 0.52 | 1.29 | 0.394 |  |  |  |  |  |
|  | 3.57-4.71 | 1.03 | 0.65 | 1.61 | 0.911 |  |  |  |  |  |
|  | 4.72-6.15 | 0.68 | 0.42 | 1.08 | 0.101 |  |  |  |  |  |
|  | >6.15 | 0.22 | 0.13 | 0.38 | <.0001 |  |  |  |  |  |
| Associated herd with an episode in current/previous year | No | Referent |  |  | . |  | Referent |  |  | . |
|  | Yes | 2.54 | 1.26 | 5.17 | 0.009 |  | 2.31 | 1.17 | 4.58 | 0.016 |
| Any reactor ever present in a previous breakdown | No |  |  |  | . |  |  |  |  |  |
|  | Yes | 3.14 | 2.26 | 4.40 | <.0001 |  |  |  |  |  |
| Ratio: No. purchased/Herd-size at the episode test | 0 | Referent |  |  | . |  | Referent |  |  | . |
|  | 0.001 - 0.0167 | 0.86 | 0.55 | 1.36 | 0.526 |  | 0.85 | 0.54 | 1.32 | 0.458 |
|  | 0.0168 - 0.0706 | 0.68 | 0.43 | 1.08 | 0.104 |  | 0.64 | 0.41 | 1.00 | 0.049 |
|  | 0.0707 - 0.2194 | 0.65 | 0.41 | 1.02 | 0.064 |  | 0.60 | 0.38 | 0.93 | 0.024 |
|  | >0.2194 | 0.49 | 0.30 | 0.78 | 0.003 |  | 0.47 | 0.30 | 0.74 | 0.001 |
| Lesion  Present in 1 or more reactors | No | Referent |  |  |  |  | Referent |  |  |  |
|  | Yes | 6.49 | 4.32 | 10.08 | <.0001 |  | 6.81 | 4.54 | 10.54 | <.0001 |
| Episode test type | 01/6 | Referent |  |  |  |  | Referent |  |  | . |
|  | 3 |  |  |  |  |  | 0.39 | 0.14 | 0.93 | 0.049 |
|  | 5 |  |  |  |  |  | 1.77 | 1.15 | 2.72 | 0.010 |
|  | 7B |  |  |  |  |  | 0.96 | 0.53 | 1.69 | 0.881 |
|  | 8 |  |  |  |  |  | 0.92 | 0.61 | 1.37 | 0.673 |
|  | 910 |  |  |  |  |  | 1.10 | 0.69 | 1.75 | 0.674 |
| Number of previous episodes in last 10 years | 0 | Referent |  |  |  |  | Referent |  |  | . |
|  | 1 |  |  |  |  |  | 0.79 | 0.54 | 1.15 | 0.225 |
|  | 2 |  |  |  |  |  | 1.07 | 0.71 | 1.60 | 0.750 |
|  | 3 |  |  |  |  |  | 1.14 | 0.66 | 1.93 | 0.641 |
|  | >3 |  |  |  |  |  | 0.32 | 0.16 | 0.60 | 0.001 |

Figure S1. Median age of reactors by the number of reactors in the breakdown

Figure S2. Minimum age of reactors by the number of reactors in the breakdown

Figure S3. Maximum age of reactors by the number of reactors in the breakdown
