# Supplementary figures and images for: Trends and Predictors of Large Tuberculosis Episodes in Cattle Herds in Ireland
Source: Front Vet Sci. 2018 May 23;5:86. doi: 10.3389/fvets.2018.00086 (PMC5974150; doi:10.3389/fvets.2018.00086)

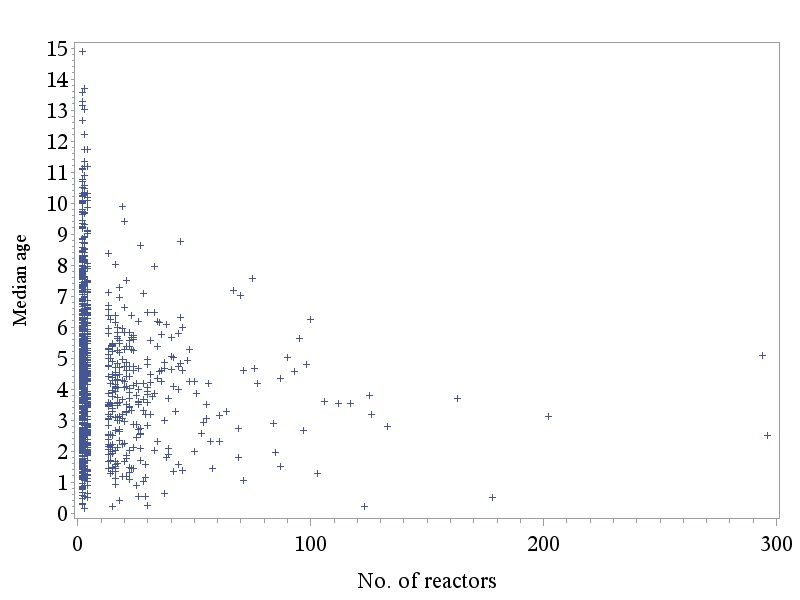

Supplement: Figure S1 — Median age of reactors by the number of reactors in the breakdown. [file Image1.JPEG]

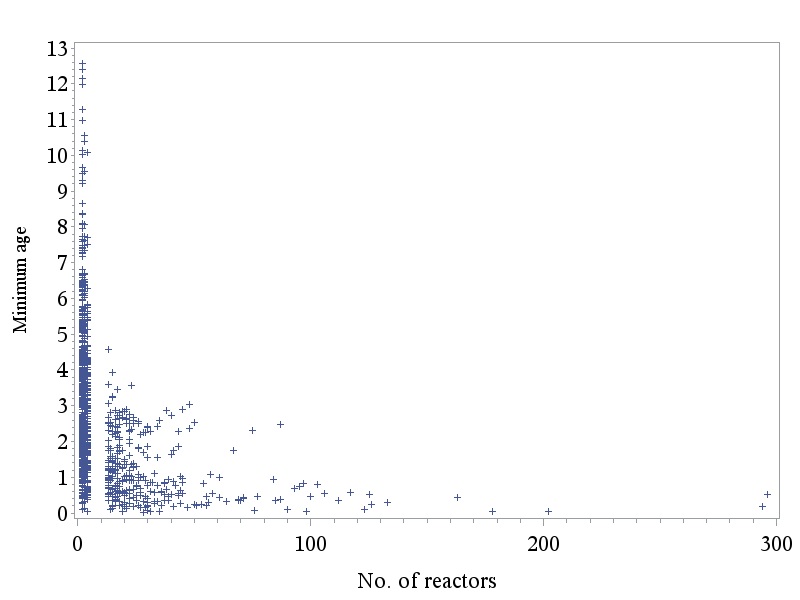

Supplement: Figure S2 — Minimum age of reactors by the number of reactors in the breakdown. [file Image2.JPEG]

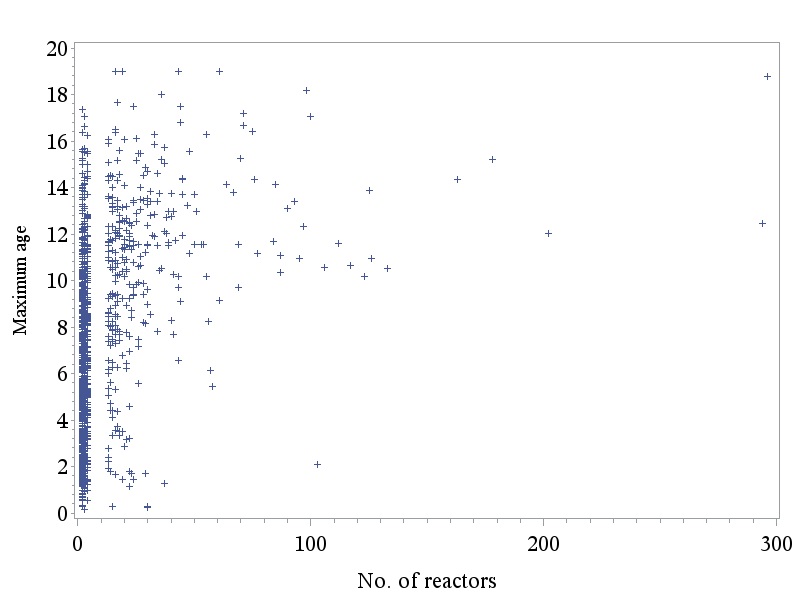

Supplement: Figure S3 — Maximum age of reactors by the number of reactors in the breakdown. [file Image3.JPEG]
